# How we teach children with asthma to use their inhaler: a scoping review

**DOI:** 10.1186/s13052-022-01237-2

**Published:** 2022-04-01

**Authors:** Patrick McCrossan, Orla Mallon, Michael D. Shields, Catherine Russell, Lesley Kennedy, Dara O’Donoghue

**Affiliations:** 1 Department of Paediatric Respiratory Medicine, Royal Belfast Hospital for Sick Children, Belfast, UK; 2 School of Medicine, Dentistry and Biomedical Sciences, Queen’s University Belfast , Belfast, UK

**Keywords:** Asthma, Inhaler, Technique, Children, Paediatrics

## Abstract

**Background:**

One reason that asthma remains poorly controlled in children is poor inhaler technique. Guidelines recommend checking inhaler technique at each clinical visit. However, they do not specify how best to train children to mastery of correct inhaler technique. Many children are simply shown how to use inhalers which results in less than 50% with correct inhaler technique. The aim of this scoping review is to explore published literature on teaching methods used to train children to master correct inhaler technique.

**Methods:**

We searched (from inception onwards): Medline, Embase, Scopus, Web of Science, CINAHL and the Cochrane library. We included quantitative studies, (e.g. randomised controlled trials, cohort studies and case-control studies), published from 1956 to present, on teaching inhaler technique to children with asthma. Data was extracted onto a data charting table to create a descriptive summary of the results. Data was then synthesised with descriptive statistics and visual mapping.

**Results:**

Thirty-three papers were identified for full text analysis. Educational interventions were found to be taking place in a variety of clinical areas and by a range of healthcare professional disciplines. ‘Brief-Instruction’ and ‘Teach-Back’ were identified as two primary methods of providing inhaler technique training in the majority of papers. Secondary themes identified were; use of written instruction, physical demonstration, video demonstrations and/or use of inhaler devices to augment inhaler technique training.

**Conclusion:**

There are a variety of means by which inhaler technique has been taught to children. These methods are likely applicable to all inhaler types and often involve some form of physical demonstration. Children of all ages can be trained to use their inhaler correctly and by a range of healthcare professionals. We have not analysed the effectiveness of these different interventions, but have described what has been trialled before in an attempt to focus our attentions on what may potentially work best. The majority of these methods can be dichotomised to either ‘Brief-Intervention’ or ‘Teach-Back’. Based on our analysis of this scoping review, we consider the following as areas for future research; how many times does a given intervention have to be done in order to have the desired effect? For what duration does the intervention need to continue to have a long-lasting effect? And, what is the best outcome measure for inhaler technique?.

**Trial registration:**

Systematic review registration: Open Science Framework (osf.io/n7kcw).

**Supplementary Information:**

The online version contains supplementary material available at 10.1186/s13052-022-01237-2.

## Background

Asthma remains poorly controlled for many children [1]. Regular inhaled corticosteroid (ICS) should provide control for the vast majority of these children [2, 3].

Effective delivery of drugs to the lungs relies on patients using their inhalers correctly [4]. However, inhaler technique; particularly in children, is generally poor [5, 6]. This results in failure to deliver an adequate dose of ICS to the airways [5]. Failure to deliver ICS to attenuate chronic airways inflammation is associated with poor asthma control, increased asthma attacks and increased risk of death [6, 7].

Inhaler technique has not improved over the last four decades despite major pharmaceutical company investment into easy to use inhaler devices [8]. Current guidelines recommend checking inhaler technique at each clinical visit [9] at which point many children are simply shown how to use their inhaler which results in less than 50% with correct inhaler technique [10]. There are many available guidelines on how to effectively use an inhaler; for example, Asthma UK have instructional videos for all of the commonly prescribed inhalers [11]. However, to the best of our knowledge, there are not yet any guidelines on how to train a child to master the technique of using their inhaler. Furthermore, there have been no previously published literature reviews exploring methods of teaching children how to use their inhaler.

The aim of this study is to review published literature on teaching the skill of inhaler technique to children with asthma. The specific objectives are:

1. To identify what different educational methods and approaches have been used to teach the skill of inhaler technique in children.
2. To consider whether these methods need to be tailored according to the age of the child.
3. To describe methods suitable for use in a children’s asthma clinic.
4. To determine how the effectiveness of these methods are evaluated.

## Methods

The protocol has been registered within the Open Science Framework platform (osf.io/n7kcw). The full protocol has also been published in BMC Systematic Reviews [12].

The study is reported in accordance with the reporting guidance provided in the Preferred Reporting Items for Systematic Reviews and Meta-Analyses Protocols (PRISMA-P) statement [13] and the PRISMA extension for Scoping Reviews (PRISMA-ScR).

### Eligibility criteria

We included studies published since the year 1956 (when the pressurised metered-dose inhaler (pMDI) was first introduced to clinical practice [14]. We included studies in which the teaching of inhaler technique was provided by doctors, nurses, pharmacists and physiotherapists. We did not wish to be ‘device specific’ and so included studies of metered-dose inhalers and dry powder inhalers, with and without the use of spacer devices. We included studies which have taken place in the emergency department, the out-patient department, the hospital ward and/or the community as these are all areas where inhaler technique teaching occurs.

We excluded studies of the use of nebulised therapies as this involves a different technique and is a more passive procedure. Studies which involve adult participants only were excluded. We did include studies which include adults and children only if the children’s data had been presented separately and could therefore be reviewed as a study of children with asthma. Publications not in English were excluded.

### Information sources and searches

**Fig. 1:**
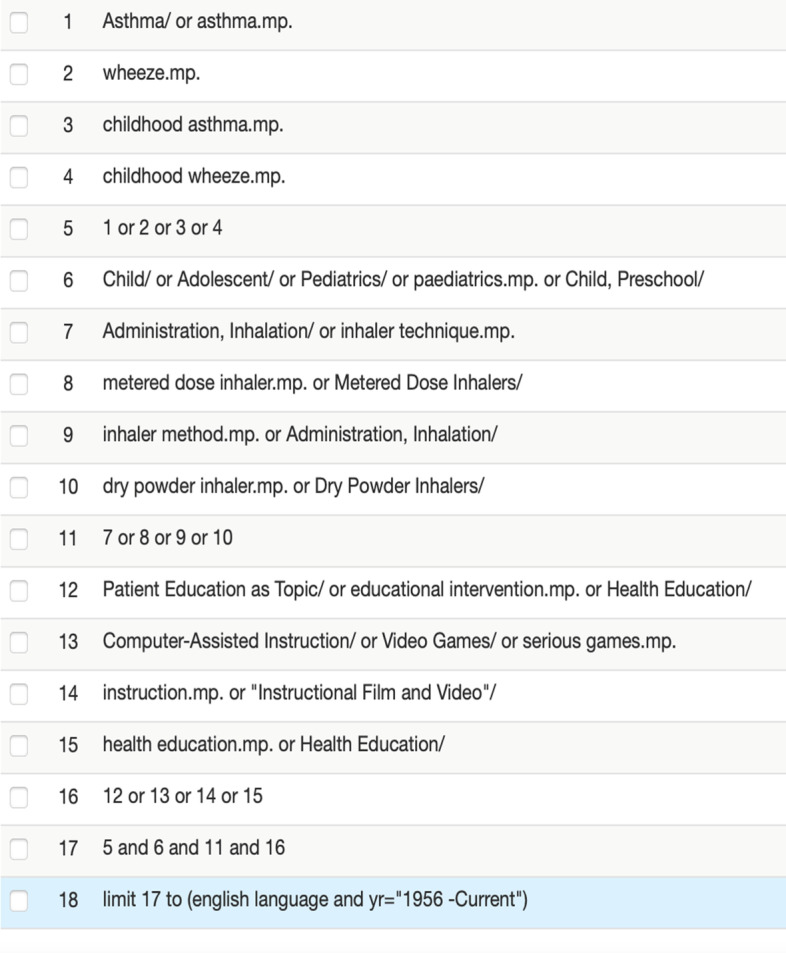
Search strategy for Medline 25/11/2021

A search on Medline, Embase, Scopus, Web of Science, CINAHL and the Cochrane library was undertaken from inception in June 2020, with the final search being completed on 26/11/2021. A search of other grey literature (including Google Scholar) and discussion with a group of paediatric asthma specialists was also undertaken to handpick any further highly relevant studies. The search strategy for Medline is found in Fig. 1. The search strategies for the other databases can be found in the appendix (Additional file 1).

### Selection of sources of evidence

**Fig. 2:**
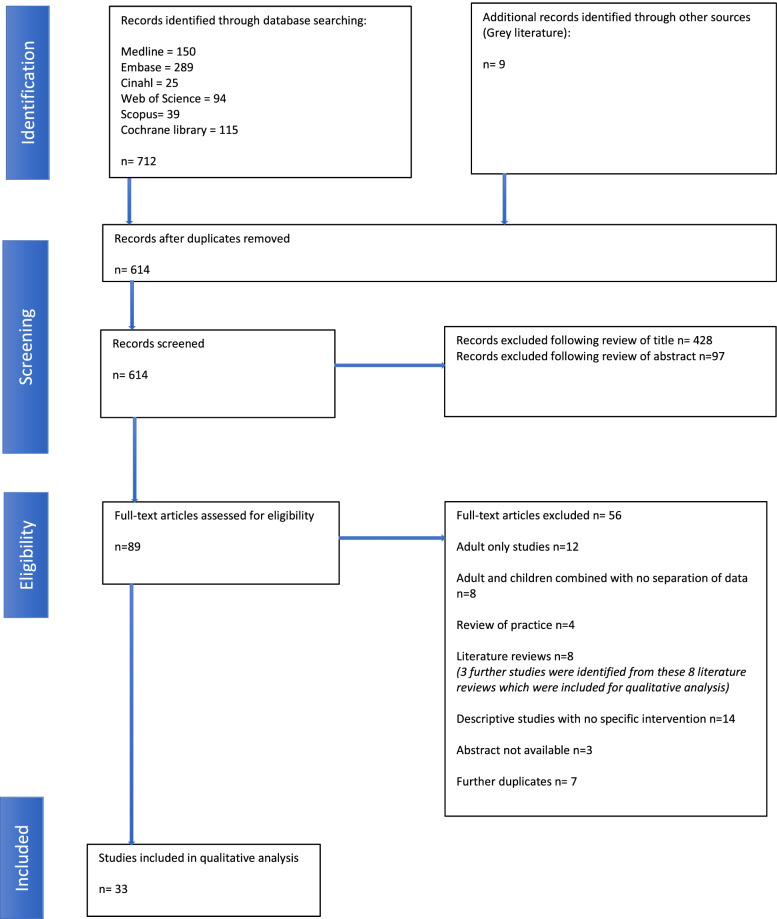
PRISMA Flow diagram

Selection of studies was performed in two stages. An initial screening of all publications based on the title with further screening based on the abstract, to ensure they fulfilled the eligibility criteria (PM). Documents not meeting eligibility criteria were excluded from full-text analysis. The remaining publications were then read by two further reviewers from the research team (OM and DO). These two reviewers independently analysed the content included in full-text articles. For cases of uncertainty, the text was re-evaluated by a third independent reviewer (MS). The final search results were exported into Endnote™ at which point all duplicates were detected and eliminated. Full-text analysis was then performed by a wider team (PM, OM, DO, MD, CR and LK). A flow chart showing details of studies included and excluded at each stage of the study selection process is seen in Fig. 2.

### Extracting and charting the data

A data extraction form was designed and used to extract equivalent information from each study report. Subsequently, each of the included studies were abstracted by two reviewers, independently, and potential conflicts were resolved through discussion.

#### Data synthesis

The data was summarised in a descriptive (narrative synthesis) and visual format (mapping summary). The strategy for data synthesis entailed the use of qualitative methods to categorise the educational interventions based on the treatment modality as well as subgroup diagnosis and age group e.g. pre-schoolers aged ≤5 years old, as they are often distinct in the literature with regards phenotype but also present different challenges with regards inhaler technique compliance [15]. Any commonalities between studies were synthesised and presented. A qualitative descriptive synthesis of data was undertaken in mapping the intervention modalities.

## Results

### Selection of sources of evidence

Figure 2 shows the study selection process. The initial search yielded 712 articles. Articles were screened first by title then by abstract. Following this process a total of 33 articles were included for data analysis. A list of included full text articles along with completed data extraction form is found in the appendix (see Additional file 2).

### Characteristics of sources of evidence

A large proportion of the studies were conducted in the USA (*n* = 12) with at least one study emanating from 12 other countries. Seventeen studies were published within the last 5 years, with no studies found before 1995.

The majority of the papers report prospective interventional studies with more than half randomised controlled trials (RCT). Three of the studies reported retrospective data.

## Results of individual sources of evidence

Fifteen of the studies used pMDI with only four using DPI (in 14 of the studies, the type of inhaler used was not specified).

The inhaler technique training was provided by a range of people, including lay persons. In 13 of the studies, training was provided by allied health care professionals^1^ and in only two of the studies was the training provided by a doctor.

There was a wide range of ages included in the studies identified. The majority (*n* = 22) were within the 5–18 year age range and therefore excluded ‘pre-schoolers’. Seven of the studies included all under 18 years and only three were exclusively in the under 5’s (in one study, the age range was not specified but stated that it was a study of children only).

The most common means of measuring inhaler technique was by using a standardised checklist (*n* = 22). Other methods included peak inspiratory flow (PIF) (*n* = 2), used as a surrogate marker of good technique and the deposition of drug on filter paper (*n* = 1). In eight of the studies the means by assessing inhaler technique was not specified.

**Fig. 3:**
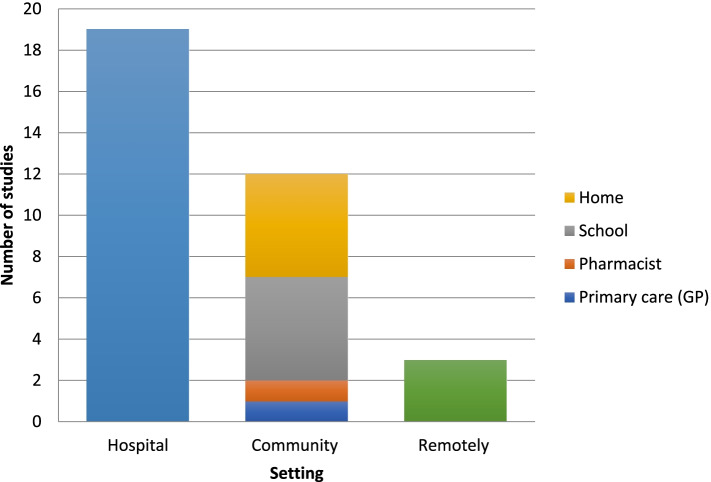
Where does inhaler training take place?

The setting of where the inhaler training took place varied throughout the studies with over half taking place within the hospital (*n* = 19), 12 in the community and in three of the studies the training was performed remotely (see Fig. 3).

### Synthesis of results

#### Emerging concepts

**Fig. 4:**
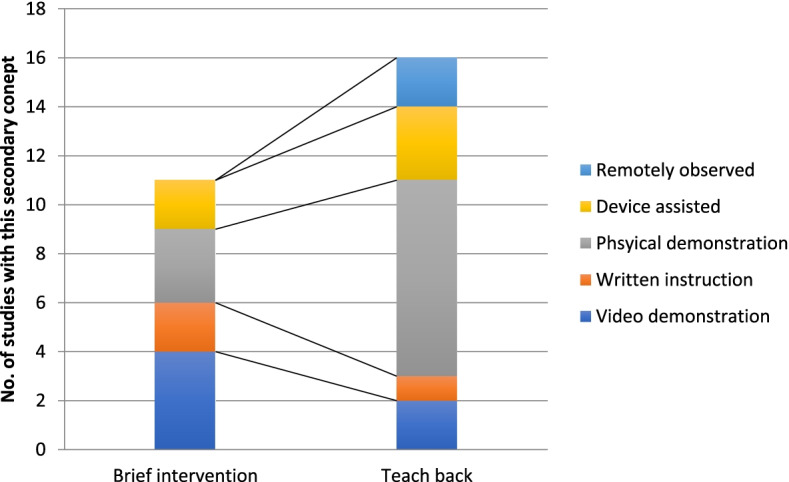
Breakdown of the number of studies with secondary concepts within the primary concepts of ‘Brief-Intervention ‘and ‘Teach-Back’

We have broken each of the studies down into Primary and Secondary concepts of educational interventions used to improve inhaler technique in children. Twenty-eight of the studies could be dichotomised to either Brief-Intervention or Teach-Back as Primary concepts. Secondary concepts (some studies included a combination of these themes) were identified as Video Instruction, Written Instruction, Physical Demonstration, Device Assisted and Remotely Observed Therapy. See Fig. 4 for a description of how these primary and secondary concepts worked together in the studies identified.

## Discussion

Inhaler technique has been proven to be inadequate in children’s asthma [10, 16] and is associated with poor asthma outcomes, including death [17]. Optimal pharmacotherapy is predicated on children performing a complicated series of steps correctly and in a proper sequence to ensure optimal therapeutic effect [10]. Therefore, proper use of an inhaler (either pMDI or DPI) is not a straightforward task and requires substantial time and effort in order to master the technique. Unfortunately, at the point at which inhalers are commonly prescribed in children (primary care and in the emergency department), this time and expert teaching is not afforded.

### Educational methods used

#### Primary concept

In her paper published in 2012, Valerie Press [18] describes ‘Brief-Intervention’ as a means of teaching a patient how to use their inhaler by providing verbal and written instruction. This is considered the most basic, least time consuming and most common means by which patients are educated to use their inhaler. This was compared with a more complex and timely strategy named ‘Teach-To-Goal’ (TTG) which “employs instruction followed by patient ‘Teach-Back,’ then repeated iterative cycles of learning and assessment until a skill is mastered.” They showed a significant improvement in inhaler technique by using this TTG strategy, however, this study did not include children. While the Press group described quite a specific method by which to provide TTG training, the essence of the method is in the Teach-Back aspect of it. That is, a patient has not proved that they have adequate technique until they have physically demonstrated this back to their instructor.

On analysing the included studies for this scoping review, we noted that many of the studies employed a strategy that had elements of Teach-Back without explicitly being named Teach-To-Goal and so we considered this a primary concept by which inhaler technique could be taught to children. As with the Press paper, the other half of included studies had methods, which were very basic and essentially aligned with her definition of ‘Brief-Intervention’. Root et al. [19] and Volerman et al. [20] are the only two that mention TTG specifically but neither are in preschool children.

##### Psychological basis of teach back

There appear to be three key processes at the core of Teach-Back; information retrieval, feedback and ‘spaced learning’. In cognitive psychology it is recognised that re-testing and retrieval, as demonstrated by Teach-Back are important to consolidate learning [20]. Study and testing form separate memories and this aids retrieval and long- term retention. Disappointingly this was reported nearly 20 years ago and yet poor inhaler technique still persists [21]. The provision of feedback helps to expedite and close the learning cycle and it is recognised that feedback-driven metacognition improves ultimate task performance [22]. The effectiveness of spaced repetition in creating long-term memories has been experimentally demonstrated in many species showing the value of spaced practice (many short sessions) over massed practice (a single long session) in long-term memory [23, 24]. Repeated stimuli separated by timed spaces without stimuli can initiate long-term memory encoding [25].

##### Precision medicine

‘Precision medicine’ has become a buzzword within medicine. The European Respiratory Journal have provided their definition within the context of asthma focusing on the stratification of patients [26]. The application of precision medicine within asthma mostly focuses on the heterogeneity of the patients and their response to specific treatments (corticosteroids and biologics etc). The same approach should therefore be used when determining how best to teach these patients how to use their inhaler device.

For example, TTG [19, 20] is a type of precision medicine as the teacher’s feedback is tailored to that specific patient’s needs. The paper by Carpenter et al. [27] regarding use of video cartoons is a perfect example where the cartoon made is bespoke to that specific child.

#### Secondary concept

While we split the primary concepts into two mutually exclusive categories, there was overlap within the secondary concepts (i.e. a number of studies used more than one secondary concept). The most common secondary concept, unsurprisingly, was the use of physical demonstration to teach inhaler technique to children. However, this was often supplemented or enhanced by other secondary concepts such as the use of inhaler devices, videos, written instructions or by performing the demonstration remotely (via video-call).

On further analysis of the different means of using videos, some of the studies used a standard video (i.e. the same instructional video shown to all participants) while others developed ‘bespoke’ videos tailored to the patients specific learning needs.

There were a number of studies which used a device. These were mostly in the form of a simple placebo or a peak flow attachment which was used to train the user to the achieve adequate flow required to use DPIs (and were therefore device- specific). There was an ‘incentive’ device named the Funhaler [28] that facilitated an improvement in inhaler technique for very young children with the benefit plateauing by 4 years old.

It was interesting that the use of remotely directly observed therapy (using video calls) had actually been studied 20 years ago [29, 30] but has been enhanced with improving technology over the years as seen in the Shields paper from 2017 [31].

### Type of inhaler used

There are two main types of inhaler, pMDIs and DPIs. The decision upon which of these is prescribed is a clinical one and at the discretion of the clinician. The method by which these inhalers are to be used is described in an instructional leaflet provided with each inhaler. The purpose of this scoping review was not to search for studies which could improve on these instructions (as they are inherent to each inhaler type) but rather to search for studies which helped patients improve their ability to follow these set of instructions. The results of our search yielded papers which were either non-device specific or specifically of pMDIs, with only 3 (of 28) specifically studying DPIs. The high number of papers using pMDIs reflects their popularity as the ‘core inhaler device’ globally [32].

It is important to mention that none of the studies specifically included use of a spacer device (despite this being included as a search term). It is best practice for children to be prescribed a spacer device alongside their pMDI, as this is proven to improve drug deposition to the lungs [9]. However, the studies included in this scoping review provide methods of teaching inhaler technique which are generic and could be feasibly used for all inhaler types including the use of spacers.

### Ages of participants

Patients perform more errors in inhaler technique at the extremes of age [33].

For paediatric patients, previous studies have shown that inhaler technique tends to improve beyond pre-school age only to dip again coming into adolescence and teenage years [34]. This scoping review identified three papers which exclusively studied pre-school children. Agertoft et al. [35] showed that improvements in technique can be made in younger children but is more difficult the younger they are. Schultz et al. [28] showed that improvements in technique can be made in younger children but that their intervention was more beneficial up until the age of 4 years, after which point it was still of benefit but not more than conventional training. The paper from Shaw et al. [36] was a study on 2–7 year-olds which showed improvements can be made in younger children and that increasing age correlates with improved technique.

There were seven studies which included (but not exclusively) preschool children. Not all of these studies attempted to stratify or compare between different ages [37–40]. Khan et al. [41] stated that “increasing age correlated directly with improved technique.” Shields et al. [31] stratified into 2–5,5–12,> 12 year olds, however this was a feasibility pilot study and so comparison between age groups was not made.

When designing educational interventions to improve inhaler technique in the future, it would be important to consider that there are distinct challenges within the different age groups of children.

### Who provided the training and where?

Our findings show that there is a broad range of health care professionals involved in the provision of inhaler training. Interestingly, it was the doctors who were least involved in actually providing the inhaler training despite the fact that in most circumstances they will be the team-member making the decision as to whether the child should be prescribed the medication. There was also a wide range of locations of where inhaler training was taking place with only half in the traditional hospital setting and a few being provided remotely.

These findings broaden the scope by which, as a community, we can improve our ability to teach children how to use their inhalers, as it has been shown to be feasible in such a diverse number of ways. ‘Not every patient is the same’ is equally true regarding their suitability for an educational intervention as it is to their specific disease process and how they respond to certain treatments.

It is beyond the scope or purpose of this scoping review to compare the efficacy of each of the included studies. However, all of the studied interventions proved feasible and showed some degree of benefit in inhaler technique. As already discussed, not all of the interventions were designed to take place in the traditional hospital clinic but rather be implemented at a variety of possibly more convenient locations such as at home or in the patient’s local community.

### Method used to measure inhaler technique

There were different means by which investigators chose to measure the effect that their educational intervention had on inhaler technique. The most commonly used was by using a standardised checklist of approved ‘steps’ which were used to score pre and post intervention. Rather than specifically counting each individual step, some researchers stratified technique based on whether the patient performed what was considered the ‘critical steps’ or categorised their ability into an overall ‘level’ of competence.

Using a defined set of steps by which to use as an ordinal measure would seem to be initially the most appealing as it takes away the bias of subjectivity. However, this may need to be balanced by the fact that many of the prescribed steps may have limited effect on drug deposition and teaching should be designed to have its biggest impact on the most clinically important aspects of inhaler use. In that sense, the studies which used PIF as a surrogate marker of good technique are also appealing [35, 42].

One of the most important aspects to teaching inhaler technique to children is not just the ability to perform correctly the technique on the day of the consultation but to sustain this ability for a prolonged period of time until mastery is achieved. Three of the studies included in this scoping review reported the sustained effect which their intervention had on inhaler technique [36, 41, 43]. Khan et al. [41] studied the effect of a DVD recording of the patient performing with good technique versus physiotherapist delivered teaching in a clinic, showed an improvement in technique sustained at 3 months (despite no difference initially). Shaw et al. [36] showed that video demonstration along with physical demonstration immediately improved technique on the first visit (compared with just physical demonstration). This difference in improvement was not sustained on subsequent follow up at 9 months. However, overall technique did improve on follow-up and so it was likely that it was the physical demonstration rather than the repeated video that sustained technique. In the paper by Sirimontakan et al. [43], technique improvement was sustained at 3 months with the introduction of a cartoon video demonstration.

### Study limitations

We acknowledge that this study has been limited to those publications in the English language; however, the broad nature of the research question still allows us to capture a significant proportion of the available literature.

Clearly the best way to prove the efficacy of a new technique (including a teaching technique) is by comparing its effect against a control. By that measure, it is reassuring that half of the studies included for analysis were of randomised controlled trials (RCT). Many of the other studies included were observational studies which helps provide the basis and rational for future work. Whilst there is a lot to be gained from other methodologies, future studies attempting to provide definitive evidence of the best means to teach children to use their inhaler correctly should ideally follow RCT methodology.

## Conclusion

Performing this scoping review has brought to our attention a wide range of educational interventions previously used to attempt to improve inhaler technique in children. We have not analysed the effectiveness of these different interventions, only described what has been trialled before in an attempt to focus our attentions on what may potentially work best going forward and get a sense of what particular methodologies or study design should be considered for future research.

The findings from this study indicate that the majority of educational interventions, despite being trialled on specific inhaler types, are likely applicable to all inhaler devices (and adjuncts) as they are generic in nature. In fact, it is likely that these same educational interventions would be appropriate for many other clinical therapies which involve the administration of medication in children.

Any member of the multi-disciplinary team can provide inhaler technique training following the requisite teaching. This has far reaching consequences as it maximises the ability of an inhaler prescriber to ensure that the patient receives adequate training when they themselves do not have the time to provide this training.

All ages of children can be taught how to use an inhaler device however it needs to be recognised that the youngest children and those entering early adulthood may find have more difficulty in initially learning and maintaining this skill. Therefore, a personalised, tailored approach must be considered, particularly when dealing with these age groups.

While not always explicitly stated, the majority of included studies used a ‘Teach-Back’ method of teaching inhaler technique as opposed to simple ‘Brief-Intervention’. Teach-Back is more time consuming but results from adult studies suggest that it is much more effective.

Some form of physical demonstration remains highly prevalent in the studies yielded by this search. The physical demonstration is often augmented by use of written instructions, devices such as placebo inhalers, video demonstrations and even remotely delivered teaching via online video-calls. This last example could become more important since the Covid-19 pandemic and the drive to provide clinical services remotely as much as possible.

The evaluation of correct or ‘mastery’ of inhaler technique is another outstanding issue which needs to be resolved as there is no clear definition and none of the studies used the exact same method.

### Implications and recommendations

All of the above mentioned techniques have been facilitated within the remit of both primary care and secondary care asthma clinics even if the training itself does not happen in the actual ‘clinic room’. Based on our analysis of this scoping review, we consider the following as areas of importance for future research: What is the best outcome measure for inhaler technique in children and how should this be defined? Is ‘Teach-Back’ more effective than ‘Brief-Intervention’ for teaching inhaler technique to children? Can the same intervention be used with equal efficacy in children of all ages? How many times does a given intervention have to be completed in order to have the desired effect? For what duration does the intervention need to continue to have a long lasting effect?

## Supplementary Information

**Additional file 1.** Exact search strategy for Embase, Web of Science, Scopus, Cinahl plus, Cochrane database registry of trials and ‘Grey literature’.

**Additional file 2.** Completed data extraction form.

## Data Availability

All data generated or analysed during this study are included in this published article [and its supplementary information files].

## Abbreviations

ICS: Inhaled Corticosteroids
PRISMA: Preferred Reporting Items for Systematic Reviews and Meta-Analyses
pMDI: Pressurised Metered-Dose Inhaler
DPI: Dry Powder Inhaler
PIF: Peak Inspiratory Flow
TTG: Teach-To-Goal
NRAD: National Review of Asthma Deaths

## Footnotes

1 Allied health professional is a non-doctor health care professional. In many of the studies a team of allied health professionals including asthma nurse specialists, physiotherapists and pharmacists were used to provide the inhaler training.
