# Supplementary material for: How we teach children with asthma to use their inhaler: a scoping review
Source: Ital J Pediatr. 2022 Apr 1;48:52. doi: 10.1186/s13052-022-01237-2 (PMC8972732; doi:10.1186/s13052-022-01237-2)
Supplement: Supplementary file 1 — Additional file 1. Exact search strategy for Embase, Web of Science, Scopus, Cinahl plus, Cochrane database registry of trials and ‘Grey literature’. [file 13052_2022_1237_MOESM1_ESM.docx]

Exact search strategy for Embase, Web of Science, Scopus, Cinahl plus, Cochrane database registry of trials and ‘Grey literature’

**Embase** 01/06/2020

1. Asthma or childhood asthma or wheeze or childhood wheeze. 2.Children or child or pediatrics or paediatrics. 3. Metered dose inhaler technique or dry powder inhaler technique or inhaler management or inhaler method or inhalational drug administration*. 4. Patient education or health education or educational intervention or patient instruction or instruction or video game or serious game or computer assisted instruction.

Searched: 1 and 2 and 3 and 4. Limited to English. 1956-present.

**Web of science** 01/06/2020

1. Asthma or childhood asthma or wheeze or childhood wheeze 2. Children or child or pediatrics or paediatrics. 3. Metered dose inhaler technique or dry powder inhaler technique or inhaler technique or inhaler management or inhaler method or inhaler adminstration 4. Serious games or inhaler instruction or patient training or educational intervention.

Searched: 1 and 2 and 3 and 4. Limited to English language. 1970- present (WOS only starts from 1970).

**Scopus** 01/06/2020

1. Asthma or childhood asthma or wheeze or childhood wheeze. 2. Children or child or pediatrics or paediatrics. 3. Metered dose inhaler or dry powder inhaler or inhaler technique or inhaler management or inhaler method or inhaler administration 4. Serious games or inhaler instruction or patient training or educational intervention.

Searched: 1 and 2 and 3 and 4. Limited to English language. 1956-present.

**CINAHL plus** 01/06/2020

1. Asthma or childhood asthma or wheeze or childhood wheeze or wheezing. 2. Children or adolescents or youth or child or teenager* or paediatrics or children or child or young person* or paediatrics. 3. Inhaler technique or inhaler method or inhaler management * or inhaler administration or metered dose inhaler technique or dry powder inhaler technique 4. Inhaler technique education* or inhaler instruction or serious games in education* or educational intervention or education or training.

Searched: 1 and 2 and 3 and 4. Limited to English language. 1956-present.

**Cochrane database registry of trials**

Search = asthma OR wheeze, children OR paediatrics OR pediatrics, inhaler technique OR inhaler method, educational intervention OR instruction OR serious games:

**Grey literature**

Using the same search terms as above we completed further searches of the following registries; *NIHR academy complete awards, ISRCTN registry,* *Clinicaltrials.gov (US national library of medicine),* *UK clinical trial gateway*, *KingsFund* and *Prospero.*
