# Supplementary material for: How we teach children with asthma to use their inhaler: a scoping review
Source: Ital J Pediatr. 2022 Apr 1;48:52. doi: 10.1186/s13052-022-01237-2 (PMC8972732; doi:10.1186/s13052-022-01237-2)
Supplement: Supplementary file 2 — Additional file 2. Completed data extraction form. [file 13052_2022_1237_MOESM2_ESM.docx]

| Paper no. | 1(1) |
| --- | --- |
| Author | Ammari W |
| Year | 2017 |
| Title | Mastery of pMDI technique, asthma control and quality-of-life of children with asthma: A randomized controlled study comparing two inhaler technique-training approaches. |
| Country of origin | Jordan |
| Aims | Compare the pMDI VC (verbal counseling) with the novel Trainhaler device tool in 7-17 year-old children with asthma attending respiratory outpatient clinics. |
| Studied population (including age range) | 7-17 year olds with asthma (already prescribed ICS). |
| Type of inhaler studied | pMDI |
| Methodology | Investigational, parallel-grouped, controlled randomised study.  Two clinic visits; with 6-8 week gap between. At the first clinic there was a performance of baseline inhaler technique scored against a standard 11-step process and peak inspiratory flow (PIF) through a pMDI measured using In-Check Flow Meter. The training of the VC and TH children at visit 1 continued until they adequately demonstrated the correct pMDI technique including a PIF <60 L/min. At the second study visit (follow up), the children demonstrated their pMDI technique using the placebo inhaler and their PIF through the pMDI was measured again. |
| Intervention type | Compare the effect of verbal counselling on pMDI training with that of a novel ‘trainhaler’ tool. |
| Who provided the inhaler training? | Not specified |
| Concept | Teach back  Inhaler device (to measure PIF as marker of good technique). |
| Measured outcomes | pMDI technique at 2^nd^ clinic (following intervention).  PIF through placebo inhaler at 2^nd^ clinic (following intervention). |
| Key findings | PIF was improved by both interventions, compared with the control group. This improvement was seen most in the VC group.  The number of technique errors was significantly reduced for both the VC and TH group between visits. |

| Paper no. | 2 (2) |
| --- | --- |
| Author | Agertoft L |
| Year | 1998 |
| Title | Importance of training for correct Turbuhaler use in preschool children. |
| Country of origin | Denmark |
| Aims | To assess whether or not training and experience improves the peak inspiratory flow rates of young children who are generally considered incapable of consistently producing effective inspiratory flow rates. |
| Studied population (including age range) | Pre-school children |
| Type of inhaler studied | DPI |
| Methodology | Prospective randomised control trial, single-blind, paralell-group study.  Stratified by age (3-4, 4-5, 5-6 year olds).  Group A received training at hospital and at home. Group B received training at hospital only. PIF was measured for all at baseline and at subsequent clinics. There were multiple educational interventions including use of videos and physical demonstrations. |
| Intervention type | There were multiple educational interventions including; 1. Three minute long video demonstration of correct use of Turbuhaler DPI with a ‘teach back’ aspect 2. Instructional pamphlet (7 steps with diagrams) 3. Individual physical training (same as video with ‘teach back’ aspect) 4.Home training using a placebo turbuhaler. |
| Who provided the inhaler training? | Not specified. |
| Where did the training take place? | Hospital and at home. |
| Concept | Teach back  Video demonstration.  Physical demonstration.  Written instruction.  Device assisted (placebo) |
| Measured outcomes | Change in PIF through Turbuhaler (following intervention). |
| Key findings | Effect of individual training on both groups showed a statistically significant improvement in PIF for children 4 years or older. |

| Paper no. | 3 (3) |
| --- | --- |
| Author | Arumugom A |
| Year | 2017 |
| Title | A Randomized Comparison between Video Demonstration and Verbal Instruction in Improving Rota Haler Technique in Children with Persistent Asthma: A Pilot Study. |
| Country of origin | India |
| Aims | To compare the technique of rota haler use in children with persistent asthma immediately after receiving either verbal instruction or a video based demonstration and again at one month following intervention. |
| Studied population (including age range) | Children >6 years old with asthma (prescribed rotahaler for first time and therefore naïve to the technique). |
| Type of inhalerr studied | DPI |
| Methodology | Pilot, open labeled randomized control trial.  Patients randomized to either verbal or video instruction. Following their inhaler instruction* they were asked to demonstrate technique and scored.  *There was limited detail regarding what was involved in the verbal instruction compared with video, only that they were standardized. |
| Intervention type | Control group (verbal instruction) v ‘video aided’. |
| Who provided the inhaler training? | Not specified. |
| Where did the training take place? | Hospital |
| Concept | Brief intervention.  Video demonstration. |
| Measured outcomes | Scores improved more in the intervention group (video) both immediately and at 1 month post intervention. |
| Key findings | Inhaler technique improved with use of video demonstration. |

| Paper no. | 4 (4) |
| --- | --- |
| Author | Martin M |
| Year | 2015 |
| Title | Results from a community-based trial testing a community health worker asthma intervention in Puerto Rican youth in Chicago. |
| Country of origin | USA |
| Aims | Efficacy of a community health worker (CHW) intervention to improve use of inhaled corticosteroids (ICS) and reduce home asthma triggers in Puerto Rican youth in Chicago. |
| Studied population (including age range) | Children between the ages of 5–18 years with persistent asthma who were attending a school-based community clinic. |
| Type of inhaler studied | Non device-specific |
| Methodology | Randomized controlled trial design.  An attention-control group received a written version of the asthma content on the same schedule as the intervention group (CHW). CHW arm were offered four CHW visits over four months in the home. CHWs used lung models and demonstrated metered dose inhalers. Participants in the attention-control group received four single-page bilingual color newsletters that covered the same core asthma topics and self-management skills.  Outcomes were assessed by research assistants blinded to study arm at pre-randomization, five months (immediately after intervention completion) and 12 months post randomisation to determine sustainability. |
| Intervention type | Individual physical demonstration of inhaler technique at home. |
| Who provided the  inhaler training? | Community health workers (trained lay persons). |
| Where did the training take place? | Home |
| Concept | Brief intervention.  Physical demonstration. |
| Measured outcomes | Number of correctly performed inhaler technique steps following intervention. |
| Key findings | Intervention (CHW) resulted in improved inhaler technique. |

| Paper no. | 5 (5) |
| --- | --- |
| Author | Carpenter D |
| Year | 2016 |
| Title | Using Tailored Videos to Teach Inhaler Technique to Children With Asthma: Results From a School Nurse-Led Pilot Study. |
| Country of origin | USA |
| Aims | Test whether a tailored inhaler technique video intervention: (1) could be feasibly implemented by school nurses and (2) improve the inhaler technique of children with asthma. |
| Studied population (including age range) | School children 7-17 years old with asthma and px MDI. |
| Type of inhaler studied | pMDI |
| Methodology | School nurses who had volunteered to participate underwent training in teaching inhaler technique.  On the 1^st^ visit with the nurse, participating children demonstrated their pMDI technique and were scored. This information was put into the video software to generate a patient specific tailored video which highlighted individuals’ previous errors. The children then watched this video (cartoon) which demonstrated the desired technique (1-2minutes). They then demonstrated their own inhaler technique immediately after watching the video. 1 month later, the children returned and demonstrated their technique again, with nil intervention between times. |
| Intervention type | Video instruction |
| Who provided the inhaler training? | School nurses (who were taught how to teach inhaler technique). |
| Where did the training take place? | Hospital |
| Concept | Video instruction |
| Measured outcomes | MDI technique was assessed using an 8-point checklist. |
| Key findings | All participating children showed an improvement in inhaler technique following intervention. |

| Paper no. | 6 (6) |
| --- | --- |
| Author | Chan D |
| Year | 2003 |
| Title | An Internet-based store-and-forward video home telehealth system for improving asthma outcomes in children. |
| Country of origin | USA |
| Aims | The purpose of this study was to develop an Internet-based store-and forward video monitoring system for patients with asthma, demonstrate its use in pediatric patients, assess a range of outcome measures, and determine whether virtual follow-up over the Internet can be effective for managing asthma. |
| Studied population (including age range) | 6-17 year olds with asthma. |
| Type of inhaler studied | Non device-specific |
| Methodology | Randomised to; 1. Internet based education 2. Office based ‘traditional’ education.  Each recruited patient received a home computer with video camera/microphone and internet cable access. An initial evaluation was performed including pulmonary function testing (PFT). Patients used the digital camera to record peak flow (reading out the measurement) and their daily use of inhaler, two times per week. A daily asthma symptom diary was also kept. Twice per week, the ‘case manager’ reviewed the videos of patients’ peak flows and inhaler use. Patients technique was scored and e-mail feedback was then given.  This intervention continued over a 6 month period. |
| Intervention type | Video recording of inhaler use with email feedback. |
| Who provided the inhaler training? | The ‘case manager’. |
| Where did the training take place? | Home |
| Concept | Remotely observed  Physical demonstration |
| Measured outcomes | Patients adherence to treatment and disease control including inhaler technique. |
| Key findings | Inhaler technique improved with video intervention. |

| Paper no. | 7 (7) |
| --- | --- |
| Author | Grover C |
| Year | 2015 |
| Title | Medication education program for Indian children with asthma: A feasibility study. |
| Country of origin | India |
| Aims | The aim of this study was to develop, implement, and evaluate the efficacy of a culturally contextualized asthma education program designed for Indian parents and children with asthma. |
| Studied population (including age range) | 7-12 year old children with asthma attending an asthma clinic. |
| Type of inhaler studied | DPI and pMDI |
| Methodology | Randomised control trial with ‘repeated’ measures.  The parent/child was randomised to usual care or the interventional program. The three key structural components of the program included: (1) A PowerPoint presentation™ (2) A child workbook and (3) Related activities interspersed at appropriate spots*. In the intervention group, when the PowerPoint presentation got to the point of discussing medications, the child demonstrated their inhaler technique and this was scored (nil detail of how this was scored).  *The 3^rd^ component is not described with regards what activities are performed but it does include a ‘teach back like’ method of teaching inhaler technique (“3 step process 1. Child demonstrates 2. Educators demonstrate 3. Child re-demonstrates until appropriate). |
| Intervention type | Enhanced teaching including use of a ‘teach back’ method. |
| Who provided the inhaler training? | Not specified |
| Where did the training take place? | Hospital |
| Concept | Teach back  Physical demonstration |
| Measured outcomes | Outcomes were measured at 1 and 6 months with a validated questionnaire and inhaler technique score. |
| Key findings | An improvement in inhaler use scores was observed in all cases within the intervention group. |

| Paper no. | 8 (8) |
| --- | --- |
| Author | Horner S |
| Year | 2008 |
| Title | Improvement of Rural Children’s Asthma Self-Management by Lay Health Educators (LHE). |
| Country of origin | USA |
| Aims | To examine changes in rural children’s asthma self-management after they received the LHE delivered classes but prior to receiving the family education session. |
| Studied population (including age range) | School children with asthma in rural areas. |
| Type of inhaler studied | pMDI |
| Methodology | Randomised into ‘treatment’ or ‘attention-control’ groups.  The intervention (skills practice and problem-solving exercises) were covered in 15-minute sequential sessions (during lunch breaks), 2-3 times per week.  Asthma intervention involved a 7-step asthma self management programme including skills practice with a placebo pMDI and peak flow meters. Attention-control intervention involved generic health promotion topics and skills practice such as brushing teeth and washing hands.  Data was collected on day 1 and 12 weeks later. |
| Intervention type | Specific asthma education, verbally delivered in schools by lay persons (nil detail of content). |
| Who provided the inhaler training? | Lay health educators (who were trained in how to teach inhaler technique). |
| Where did the training take place? | School (community). |
| Concept | Brief intervention.  Use of placebo (device). |
| Measured outcomes | Asthma knowledge, asthma self-management, asthma self-efficacy and pMDI technique (using GRAs 8 item scoring system). |
| Key findings | pMDI skill improved for the ‘treatment group’. |

| Paper no. | 9 (9) |
| --- | --- |
| Author | Kamps A |
| Year | 2002 |
| Title | Determinants of correct inhalation technique in children attending a hospital-based asthma clinic. |
| Country of origin | Sweden |
| Aims | To identify factors that are associated with correct inhalation technique in children with asthma. |
| Studied population (including age range) | Children attending an asthma clinic (all ages). |
| Type of inhaler studied | MDI and DPI |
| Methodology | Patients at clinic were asked to demonstrate inhaler technique and were scored using standard check lists. Patients then completed a questionnaire which included questions regarding previous teaching of inhaler technique.  To act as a ‘reference’ group, 47 newly referred children with asthma were prospectively studied. These patients received repeated standardised instructions. |
| Intervention type | Repeated standardised instructions |
| Who provided the inhaler technique? | Either a paediatric pulmonologist or an asthma nurse |
| Where did the training take place? | Hospital |
| Concept | Teach back |
| Measured outcomes | Number of correctly performed inhaler technique steps using standard checklist. |
| Key findings | Of the patients who had received inhalation instructions at least one more time after the initial instructions, 91% performed all the essential steps correctly, compared with 62.5% of the patients who had received a single inhalation instruction at the time of prescription (p < 0.001). |

| Paper no. | 10 (10) |
| --- | --- |
| Author | Kamps A |
| Year | 2000 |
| Title | Poor Inhalation Technique, Even After Inhalation  Instructions, in Children With Asthma. |
| Country of origin | Netherlands |
| Aims | To evaluate the usefulness of inhalation instructions. |
| Studied population (including age range) | New patients referred to paediatric asthma clinic (all ages). |
| Type of inhaler studied | pMDI and DPI |
| Methodology | Patients newly referred to asthma clinic were asked to demonstrate inhalation technique (scored using standard checklist). These patients filled out a questionnaire asking about their previous inhaler instructions.  As a reference guide, inhalation technique was also scored for children who were recruited for another clinical trial (they were instructed and reviewed at least twice per week for 4 weeks). |
| Intervention type | Questionnaire of previous inhaler training experience. |
| Who provided the inhaler training? | Pharmacists |
| Where did the training take place? | Community (pharmacist) |
| Concept | Teach back |
| Measured outcomes | Number of correct inhaler technique steps completed  compared with type/length of inhaler training. |
| Key findings | Of the 29 patients who had received inhalation instruction at the pharmacy*, 23 (79%) performed all essential steps correctly, compared to only 12 of 31 (39%) of patients who had been trained by their GP (or his/her nurse) (P=.0014). This difference was independent of the type of inhaler used.  *In pharmacies, inhalation instructions were usually repeated until the patient performed satisfactorily according to the trainer; this took up to 30 min. In general practice, the training relied mostly on verbal instructions, without demonstrating the correct procedure, generally lasting no more than 5 min. |

| Paper no. | 11 (11) |
| --- | --- |
| Author | Khan R |
| Year | 2018 |
| Title | DVD v Physiotherapist-Led Inhaler Education: A Randomised Controlled Trial. |
| Country of origin | Ireland |
| Aims | Benefit of personalised video instruction v in-person instruction of inhaler technique. |
| Studied population (including age range) | Convenience sample of children admitted to paediatric ward over 3 months who were prescribed an inhaler (all ages). |
| Type of inhaler studied | Non device- specific |
| Methodology | Randomised, single blinded controlled trial.  Participants were randomly allocated to one of two groups; 1. Intervention group which received inhaler instruction via a purpose-made DVD 2. Control group which received the usual care of individual instruction from a physiotherapist.  Prior to education, the non-naïve patients were videoed using a placebo inhaler. The intervention group watched then DVD as many times as desired and practised with placebo. They demonstrated their technique on video and were given a copy of this DVD for home viewing. The control group received standard management which included scripted education from a physiotherapist (content equivalent to the DVD). The physiotherapist then demonstrated the technique followed by the participants demonstrating the technique and feedback given.  All patients were followed up 3 months later at which point technique was re-recorded. |
| Intervention type | Personalised video v in-person ‘teach back’ like method. |
| Who provided the inhaler training? | Physiotherapist |
| Where did the training take place? | At home and hospital. |
| Concept | Teach back.  Video instruction (including a bespoke, personalised video).  Device assisted (placebo). |
| Measured outcomes | Primary outcome measures were inhaler technique score derived by the authors from manufacturers’ instructions checklist. |
| Key findings | There was a statistically significant improvement in inhaler technique score immediately after education across all subjects, deteriorating slightly at three months. There were no significant between-group differences either immediately post intervention, or after three months. However, the median DVD group score was improved after three months, whereas the median score for the individual education group deteriorated. |

| Paper no. | 12 (12) |
| --- | --- |
| Author | Minai B |
| Year | 2004 |
| Title | Results of a Physician and Respiratory Therapist Collaborative Effort to Improve Long-Term Metered Dose Inhaler Technique in a Pediatric Asthma Clinic. |
| Country of origin | USA |
| Aims | Examined whether pMDI-technique improvement is sustainable and if it improves pulmonary function and asthma outcomes in one cohort of children. |
| Studied population (including age range) | Inner city children with asthma (older than 4 years of age). |
| Type of inhaler studied | pMDI |
| Methodology | Children older than 4 years of age were referred to the clinic if they had excessive emergency-department visits or were perceived as difficult to treat by their primary care providers and had been prescribed a pMDI before the visit.  Children underwent a standardized assessment of pulmonary function and MDI technique (8 steps). A respiratory therapist demonstrated and reinforced correct pMDI technique at each visit*. Data were collected at the initial patient visit and the last recorded visit.  *No specific details about how technique was taught or reinforced is described. |
| Intervention type | Enrolment in specialised clinic where specific inhaler technique was taught and re-inforced. |
| Who provided the inhaler training? | Respiratory therapists. |
| Where did the training take place? | Hospital |
| Concept | Teach back  Physical demonstration |
| Measured outcomes | PFT’s and pMDI technique scores (using a standardised checklist). |
| Key findings | Student’s t test indicated a statistically significant improvement in pMDI scores (p <0.001) but there was no statistically significant relationship between improved MDI score and pulmonary outcomes. |

| Paper no. | 13 (13) |
| --- | --- |
| Author | Patterson E |
| Year | 2005 |
| Title | A cluster randomised intervention trial of asthma clubs to improve quality of life in primary school children: the School Care and Asthma Management Project (SCAMP). |
| Country of origin | UK |
| Aims | To evaluate the effectiveness of a school-based education programme of weekly asthma clubs in improving quality of life for primary school children with asthma. |
| Studied population (including age range) | School children with asthma. |
| Type of inhaler studied | Non device specific. |
| Methodology | A cluster randomised intervention trial with schools as the unit of randomization.  Children in schools allocated to the immediate group attended asthma clubs directly after the preliminary assessment while those in the delayed group received the same intervention after a 16-week interval. Each session began with brief reinforcement of previous training and finished with end of session feedback. |
| Intervention type | Enrolment in a school based asthma education programme. |
| Who provdided the inhaler training? | Nurses. |
| Where did the training take place? | School (community). |
| Concept | Brief intervention  Device assisted (placebo) |
| Measured outcomes | The primary outcome was overall quality of life measured using the Paediatric Quality of Life Questionnaire.  Inhaler technique was investigated using inhalers at each assessment (except the preliminary assessment in the delayed group). Results were recorded on a three-point scale (correct, partially correct, or poor) based on those criteria relevant to the child’s usual device. |
| Key findings | Inhaler technique at week 16 was markedly better in the immediate group, 59 (73%) of whom had been recommended at the start of club assessment to see their family doctor or asthma nurse to obtain a spacer, to change to a more suitable device, to standardise treatment where multiple devices were being used, to consider add on therapy, or to reinforce regular therapy. |

| Paper no. | 14 (14) |
| --- | --- |
| Author | Root J |
| Year | 2019 |
| Title | Improving asthma control in children using the teach-to-goal (TTG) method. |
| Country of origin | USA |
| Aims | The intervention for this project was to ensure children with persistent asthma could achieve correct pMDI technique, thus improving asthma control. |
| Studied population (including age range) | Asthma children in an urban clinic (school-aged). |
| Type of inhaler studied | pMDI |
| Methodology | Children who were diagnosed with persistent asthma, were developmentally able to perform pMDI technique and attended all visits were included in this project.  This project was designed to include three patient visits over a 6-month period. To achieve con­sistent educational practices, the project lead delivered a brief pMDI education session to clinic staff and provided them with the 6-step pMDI handout. Respiratory care practitioners (RCPs) and nurse practitioners (NPs) were encouraged to apply the concept of the Teach-to-Goal method and include patients return demonstrate the pMDI tech­nique until correct use was achieved. |
| Intervention type | Implementation of a nurse-led TTG method of teaching inhaler technique. |
| Who provided the inhaler training? | Asthma nurses. |
| Where did the training take place? | Hospital. |
| Concept | Teach back (Teach to goal).  Physical demonstration. |
| Measured outcomes | Asthma quality of life questionnaire and number of correct steps for inhaler technique. |
| Key findings | Significant improvement in inhaler technique following TTG. |

| Paper no. | 15 (15) |
| --- | --- |
| Author | Shaw N |
| Year | 2016 |
| Title | Pressurised metered dose inhaler-spacer technique in young children improves with video instruction. |
| Country of origin | Australia |
| Aims | To determine which steps in pMDI-spacer technique are most often performed incorrectly in young children and, secondly, to investigate the effects of an age appropriate instructional video on proficiency in pMDI spacer technique in young children. |
| Studied population (including age range) | Children with asthma who are prescribed ICS with pMDI and a spacer (this was part of a larger RCT comparing spacer devices). All ages included. |
| Type of inhaler studied | pMDI with spacer. |
| Methodology | At a screening visit, technique was checked and corrected by the study doctor if necessary. Participants were followed-up every 3 months for 12 months. At each of the four visits, participants were asked to demonstrate their device technique with five puffs of salbutamol. A single physician investigator using an 8-step checklist assessed participants’ spacer technique. At each study visit, after the initial assessment of spacer technique, participants and their parents were shown a short, age-appropriate instructional video on correct spacer technique and asked to demonstrate their device technique again. No additional instruction in device technique was given during the course of the study. |
| Intervention type | Use of video instruction to teach inhaler technique |
| Who provided the inhaler training? | Doctor |
| Where did the training take place? | Hospital |
| Concept | Video instruction. |
| Measured outcomes | Inhaler technique scored (using 8-step standardised checklist) following instructional video and at follow up clinic visits. |
| Key findings | Immediate improvement following video (at baseline) but not at subsequent visits. However, overall technique continued to improve with successive vists (juts not necessarily followng the video). |

| Paper no. | 16 (16) |
| --- | --- |
| Author | Shields M |
| Year | 2017 |
| Title | Mobile direct observation of therapy (MDOT) - A rapid systematic review and pilot study in children with asthma. |
| Country of origin | UK |
| Aims | To determine if it is feasible to provide MDOT for children with asthma to feedback and improve inhaler technique. |
| Studied population (including age range) | Children aged 2-16 with uncontrolled asthma who were prescribed ICS. |
| Type of inhaler studied | Not specified |
| Methodology | Randomised intervention trial.  Randomised to; 1. Immediate MDOT or 2. Delayed MDOT.  Stratified to 2-5,5-12,12-16 years old.  Group 1 received MDOT for first six weeks. Group 2 had a six-week intervention free period followed by MDOT. All subjects were trained on proper technique by a respiratory nurse using the TTG technique and received standard asthma clinic management. MDOT was used independently if the child was older than eight years. Video’ displaying the child’s use of their pMDI were uploaded twice daily and a member of the team evaluated their technique based on the video footage. If there was a concern regarding technique then they were contacted by phone to provide feedback. |
| Intervention type | Directly Observed Therapy with feedback (remote via mobile video app). |
| Who provided the inhaler training? | Asthma nurses |
| Where did the training take place? | Remotely |
| Concept | Teach back (Teach to goal).  Remote observed therapy.  Physical demonstration. |
| Measured outcomes | Inhaler technique categorised on a 3 point scale; effective, partially effective and poor. |
| Key findings | By week 4 of MDOT engagement, approximately 90% (17/19) of the children had inhaler technique which was judged as effective. By week 5 all children still enrolled in the study (n = 18) were judged to have effective inhaler technique i.e. inhaler technique improved after tailored inhalation instructions over the telephone by a member of clinical team. |

| Paper no. | 17 (17) |
| --- | --- |
| Author | Tukerli A |
| Year | 2016 |
| Title | Metered dose inhaler-spacer use education effects on achieve asthma control in children. |
| Country of origin | Turkey |
| Aims | To evaluate the change in asthma control after standardised education on proper pMDI-Spacer use. |
| Studied population (including age range) | Children with uncontrolled asthma (all ages included). |
| Type of inhaler studied | pMDI with spacer |
| Methodology | All children and/or parents were asked to demonstrate how they use their pMDI-spacer at the beginning of the study. Then, the pediatric allergy and pulmonology nurse demonstrated pMDI-spacer use to the child and parent. Finally, the child and/or parent were asked to demonstrate their pMDI-spacer use again.  Patients were brought back two months later and reassessed. |
| Who provided the inhaler training? | Nurse. |
| Where did the training take place? | Hospital. |
| Concept | Brief intervention.  Physical demonstration. |
| Measured outcomes | Asthma quality of life questionnaire.  Inhaler technique was scored using an 8-step process. |
| Key findings | Mean inhalation technique score was 4.9 ± 1.3 before education and increased significantly to 7.8 ± 0.4 after education (p< 0.001). |

| Paper no. | 18 (18) |
| --- | --- |
| Author | Christiansen S |
| Year | 1996 |
| Title | Evaluation of a school-based asthma education program for inner-city children. |
| Country of origin | USA |
| Aims | To improve asthma outcomes in children from inner city background. |
| Studied population (including age range) | 4^th^ grade student volunteers enrolled in four schools in San Diego who displayed asthma-like symptoms. |
| Type of inhaler studied | Not specified. |
| Methodology | Students were enrolled in either the educational prorgam or in a control non-educational group.  The program involved a five-session asthma education program including use of an inhaler. The lessons were 20 minutes long (during school time) over a five-week period. Classes were conducted in small groups. Students in the education group were followed up for the following year on a monthly basis. The non-intervention group did not participate in this class but were tested and followed up in the same fashion. |
| Intervention type | Classroom delivered asthma education (generic). |
| Who provided the inhaler training? | School nurse |
| Where did the training take place? | In school (community). |
| Concept | Brief intervention. |
| Measured outcomes | Multiple outcome measures, including; peak flow technique, and inhaler technique (7 steps). |
| Key findings | Peak flowmeter scores and inhaler technique scores improved All changes in this group were highly significant. |

| Paper no. | 19 (19) |
| --- | --- |
| Author | Salisbury C |
| Year | 2001 |
| Title | A randomised controlled trial of clinics in secondary schools for adolescents with asthma. |
| Country of origin | UK |
| Aims | To compare a nurse-led clinic in schools versus care in general practice for adolescents with asthma. |
| Studied population (including age range) | Secondary school children with asthma. |
| Type of inhaler studied | Not specified. |
| Methodology | Randomised control trial of a nurse-run asthma clinic in four secondary schools.  Participants were randomised to have a review of asthma at: 1. School clinics 2. GP clinic. Two control groups were included (pupils in the GP and pupils in schools). Schools were stratified by deprivation. A nurse-run asthma clinic ran once per week in the trial schools. The review was similar to that at the GP clinic except tailored for emphasis on adolescents. |
| Intervention type | Asthma-specialist nurse run clinic |
| Who provided the inhaler training? | Asthma nurses |
| Where did the training take place? | School |
| Concept | Brief intervention |
| Measured outcomes | Primary outcome included quality of life questionnaires.  Secondary outcome included inhaler technique (among others). |
| Key findings | Pupils in the school clinic group had higher inhaler technique scores compared with those randomised to GP care (P<0.001), but differences in scores varied significantly between schools. |

| Paper no. | 20 (20) |
| --- | --- |
| Author | Schultz A |
| Year | 2012 |
| Title | Incentive device improves spacer technique but not clinical outcome in preschool children with asthma. |
| Country of origin | Australia |
| Aims | The objective of this study is to investigate the influence of an incentive spacer (Funhaler), on spacer technique and clinical outcome in young children with recurrent wheeze. |
| Studied population (including age range) | 2-6 year olds with asthma. |
| Type of inhaler studied | Use of spacer with a pMDI. |
| Methodology | After a 1-month run-in period, at the baseline study visit, subjects were randomised to receive regular inhaled fluticasone through either a conventional valved spacer (Aerochamber Plus), or a Funhaler.  Subjects were followed up every three months for a year. Proficiency in spacer technique was measured at the first four visits by measuring the amount of salbutamol inhaled from spacer onto a filter interposed between subject and spacer. |
| Intervention type | Use of an incentive device to improve inhaler technique. |
| Who delivered the inhaler training? | Not specified |
| Where did the training take place? | Home |
| Concept | Inhaler (incentive) devices. |
| Measured outcomes | Amount of drug being delivered as a proxy of good technique. |
| Key findings | After correcting for age and gender, the Funhaler group demonstrated significantly higher proficiency in spacer technique as determined by filter dose. The improved proficiency in spacer technique in the Funhaler group was limited to subjects younger than 4 years of age at the time of randomisation. |

| Paper no. | 21 (21) |
| --- | --- |
| Author | Aziz NA |
| Year | 2006 |
| Title | Skills amongst parents of children with asthma: a pilot interventional study in primary care setting. |
| Country of origin | Malaysia |
| Aims | To assess the skills of inhaler technique amongst parents with asthmatic children attending a primary care clinic before and after an asthma education program. |
| Studied population (including age range) | Children with asthma (all ages). |
| Type of inhaler studied | pMDI |
| Methodology | Cross-sectional, clinic based study.  Inhaler technique scored pre and post intervention using a standardised 8 step checklist. |
| Intervention type | Written information regarding asthma self-care and a demonstration of how to use their inhaler (not expanded upon) during a 30-minute face-face consultation with the investigator. |
| Who delivered the inhaler training? | Doctor |
| Where did the training take place? | Primary care |
| Concept | Brief intervention  Written instruction. |
| Measured outcomes | Asthma knowledge and inhaler technique (scored against an 8 point checklist). |
| Key findings | The mean score for skills of inhaler technique improved significantly after educational intervention. |

| Paper no. | 22 (22) |
| --- | --- |
| Author | Bynum A |
| Year | 2001 |
| Title | The effect of telepharmacy counseling on metered-dose inhaler technique among adolescents with asthma in rural Arkansas. |
| Country of origin | USA |
| Aims | Effect of telepharmacy counselling, using interactive compressed video, on metered-dose inhaler technique. |
| Studied population (including age range) | Adolescents with asthma |
| Type of inhaler studied | pMDI |
| Methodology | Random assignment of participants to; 1. telepharmacy group 2. Control group.  Pre and post-test and 2-4 week check-ups for pMDI technique were performed. |
| Intervention type | Interactive video (VDOT) and pharmacy delivered education. |
| Who delivered the inhaler training? | Pharmacist |
| Where did the training take place? | Remotely. |
| Concept | Teach back  Remotely observed therapy  Physical demonstration. |
| Measured outcomes | Inhaler technique based on standardised checklist. |
| Key findings | Pre-test to follow up, the telepharmacy counselling group showed more improvement in pMDI technique than participants in the control group. |

| Paper no. | 23 (23) |
| --- | --- |
| Author | Foland AP |
| Year | 2002 |
| Title | Improvement of metered-dose inhaler administration technique: The effect of training sessions at a specialized pediatric asthma compliance and technique clinic. |
| Country of origin | USA |
| Aims | To determine whether a specialized Pediatric Asthma Compliance and Technique clinic could be effective in improving techniques for MDI use among children with asthma. |
| Studied population (including age range) | Children attending the clinic (all ages). |
| Type of inhaler studied | pMDI |
| Methodology | Participants referred to the clinic demonstrated their technique at baseline. They underwent coaching and were asked to demonstrate technique again. |
| Intervention type | Repeated face-face instruction and critique of inhaler technique. |
| Who provided the inhaler training? | Doctors and respiratory therapists. |
| Where did the training take place? | Hospital |
| Concept | Teach back |
| Measured outcomes | Number of correctly performed inhaler technique steps (scored using a standardised checklist). |
| Key findings | Only 12% (326) were able to perform all 8 steps for pMDI administration correctly (median 4 of 8 steps performed correctly without prompting). After the first coaching session, patients were followed up an average of 2.4 months later, with 31% (826) performing all 8 steps correctly. |

| Paper no. | 24 (24) |
| --- | --- |
| Author | Horner SD |
| Year | 2014 |
| Title | Evaluating the effect of an asthma self-management intervention for rural families. |
| Country of origin | USA |
| Aims | To present outcomes of an asthma self-management educational intervention delivered to children (grades 2–5) at school and to parents in a home visit. |
| Studied population (including age range) | School children with asthma |
| Type of inhaler studied | pMDI |
| Methodology | Schools randomised to intervention and no intervention  Data collected at 4 points, over 12 months. |
| Intervention type | Community / school and home based training.  16 sequential fifteen-minute teaching sessions which focused on asthma self-management (including inhaler technique, though the method of teaching was not specified). Also, a video demonstration of inhaler technique to be watched at home and also a written asthma action plan. |
| Who provided the inhaler training? | Not specified |
| Where did the training take place? | School and at home |
| Concept | Video demonstration.  Brief intervention. |
| Measured outcomes | Inhaler technique was scored using an 8- point standardised checklist. |
| Key findings | Significant improvements in inhaler skill and asthma severity were seen in the treatment group children when compared to the control group |

| Paper no. | 25 (25) |
| --- | --- |
| Author | Sleath B |
| Year | 2012 |
| Title | Communication during pediatric asthma visits and child asthma medication device technique 1 month later. |
| Country of origin | USA |
| Aims | This study investigated how provider demonstration of and assessment of child use of asthma medication devices and certain aspects of provider-patient communication during medical visits is associated with device technique 1 month later. |
| Studied population (including age range) | Children with asthma aged 8-16 years old. |
| Type of inhaler studied | Non device- specific. |
| Methodology | Observational study of provider-patient communication about asthma medication devices during primary care clinic visits.  Nil intervention.  Asthma clinic visits were voice-recorded. Children were interviewed one month later and their technique was scored. |
| Intervention type | Nil prospective intervention. |
| Who provided the inhaler training? | Research assistants (who were trained in inhaler technique). |
| Where did the training take place? | Hospital |
| Concept | Teach back |
| Measured outcomes | Number of correctly completed steps in inhaler technique using an standardised checklist. |
| Key findings | If the provider asked the child to demonstrate metered dose inhaler technique during the medical visit, the child was significantly more likely to perform a greater percentage of inhaler steps correctly one month later. |

| Paper no. | 26 (26) |
| --- | --- |
| Author | Volerman A |
| Year | 2019 |
| Title | A feasibility study of a patient-centered educational strategy for rampant inhaler misuse among minority children with asthma. |
| Country of origin | USA |
| Aims | Feasibility study of the teach to goal technique for training mastery of inhaler technique in children with asthma |
| Studied population (including age range) | Low-income, African-American children with asthma (all ages up to 18 years old). |
| Type of inhaler studied | pMDI |
| Who providied the inhaler training? | An asthma educator |
| Where did the training take place? | Hospital |
| Methodology | Prospective quasi-experimental study of a predominantly low-income population.  Children with physician-diagnosed asthma identified during school-based screening were recruited. An asthma educator met one-on-one for 45-60minutes with each child and their parent at the school or in the community. Participants completed questionnaires about their asthma, inhaler usage and demographics.  The asthma educator assessed the child’s baseline inhaler technique using a 12-step checklist. Then, the educator taught and assessed the inhaler technique using the TTG method. |
| Intervention type | TTG |
| Concept | Teach back (using teach to goal).  Physical demonstration. |
| Measured outcomes | Inhaler technique pre and post intervention using a standardised checklist. |
| Key findings | Comparing baseline and post-TTG scores, there was a statistically significant improvement in the proportion of children performing each step correctly. |

| Paper no. | 27 (27) |
| --- | --- |
| Author | Gracia-Antequera M |
| Year | 199 |
| Title | An intervention to improve the inhaler technique of children and adolescents with asthma. |
| Country of origin | Spain |
| Aims | Not available (abstract only) |
| Studied population (including age range) | Children attending an asthma clinic. |
| Type of inhaler studied | Not specified |
| Methodology | Participants were randomly selected from the asthma clinic.  Children were assessed on their inhaler technique using a questionnaire. Structured sessions of correct use were offered and new assessment 10 months later. |
| Intervention type | New education (nil specific details). |
| Who provided the inhaler training? | Not specified |
| Where did the training take place? | Hospital |
| Concept | Brief intervention.  Physical demonstration. |
| Measured outcomes | Number of correctly performed inhaler manoeuvres (non specific) |
| Key findings | Improvement in correct manoeuvres with any of the three inhaler devices after active performance training was observed. |

| Paper no. | 28 (28) |
| --- | --- |
| Author | Sirimontakan T |
| Year | 2019 |
| Title | Efficacy of a Newly Developed Cartoon Video Instruction to Improve Dry Powder Inhaler Technique in Thai Children with Asthma. |
| Country of origin | Thailand |
| Aims | To assess efficacy of cartoon video instruction in improvement of DPI technique in children. |
| Studied population (including age range) | Children with asthma 6-15 years old using DPI. |
| Type of inhaler studied | DPI |
| Methodology | Multi-centre study.  Patients were asked to demonstrate inhaler use. Four videos were then recorded; 1. prior to seeing paediatrician, 2. after watching the cartoon video demonstration 3. one month later and 4. three months later. Scoring of technique was done independently (scored out of 12-14, depending on inhaler type). |
| Intervention type | 2 minute cartoon video instruction. |
| Who provided the inhaler training? | Not specified |
| Where did the trainig take place? | In hospital |
| Concept | Video instruction |
| Measured outcomes | Number of correctly performed inhaler technique steps |
| Key findings | For both types of inhaler, improvement in technique was seen and sustained for 3 months. |

| Paper no. | 29 |
| --- | --- |
| Author | Jove Blanco A |
| Year | 2021 |
| Title | Impact of an Asthma Education Program During Admission |
| Country of origin | Spain |
| Aims | To evaluate the effect of an educational intervention on inhaler technique in children with asthma |
| Studied population | Children admitted to hospital |
| Type of inhaler studied | Not specified |
| Methodology | Prospective |
| Intervention type | ‘educational intervention’ |
| Who provided the inhaler training? | Not specified |
| Concept | Brief intervention |
| Measured outcomes | Evaluation of inhaler technique pre-post using check list |
| Key findings | Improvement of inhaler technique post intervention and one month later |

| Paper no. | 30 |
| --- | --- |
| Author | Azak M |
| Year | 2021 |
| Title | The effect of training of metered-dose inhaler technique on asthma control and quality of life in children with asthma: a randomised trial |
| Country of origin | Turkey |
| Aims | To determine the effect of two different training methods (Video-based and ‘brochure’) |
| Studied population | 7-11 year old children with asthma |
| Type of inhaler studied | MDI |
| Methodology | Randomised, single-blind, pretest-posttest design |
| Intervention type | Video-based teaching OR Brochure/Patient Info Leaflet |
| Who provided the inhaler training? | Researcher (not specified) |
| Concept | Video-based, Patient Info Leaflet + Brief Instruction |
| Measured outcomes | Number of correct steps based on checklist |
| Key findings | Inhaler technique improved on follow up in both groups, but the video more than the brochure |

| Paper no. | 31 |
| --- | --- |
| Author | Kapoor I |
| Year | 2020 |
| Title | Video education vs physical demonstration of inhaler technique for children with asthma: a randomized controlled trial |
| Country of origin | India |
| Aims | Evaluate efficacy of video-based inhaler technique training versus conventional physical instruction |
| Studied population | Children aged 6-11 years in asthma out-patient clinic |
| Type of inhaler studied | Not specified |
| Methodology | RCT |
| Intervention type | 2-minute instructional video |
| Who provided the inhaler training? | Not specified |
| Concept | Video-based training, Brief Instruction |
| Measured outcomes | Correct steps in inhaler technique |
| Key findings | No statistical difference at 12 week follow up between intervention (ie video based) and conventional (ie physical demonstration) |

| Paper no. | 32 |
| --- | --- |
| Author | Tony S |
| Year | 2021 |
| Title | The effect of adding a training device and smartphone application to traditional verbal counselling in asthmatic children |
| Country of origin | Egypt |
| Aims | Evaluate the effect of advanced counselling using a smartphone application and training device compared with traditional methods on frequency of mistakes in MDI inhaler technique |
| Studied population | 9-18 year olds with asthma |
| Type of inhaler studied | MDI |
| Methodology | Prospective randomised study |
| Intervention type | Training device and smartphone app |
| Who provided the inhaler training? | Not specified |
| Concept | Device, apps + brief instruction |
| Measured outcomes | Number of mistakes in inhaler technique, FEV1 |
| Key findings | Number of mistakes reduced on follow up in both groups but more so in the intervention group |

| Paper no. | 33 |
| --- | --- |
| Author | Gao G |
| Year | 2020 |
| Title | A randomised controlled trial of a nurse-led education pathway for asthmatic children from outpatient to home |
| Country of origin | China |
| Aims | To evaluate the efficacy of a nurse-led education pathway on children with asthma |
| Studied population | Children 4-14 years old with asthma |
| Type of inhaler studied | DPI |
| Methodology | Randomised controlled trial (RCT) |
| Intervention type | Nurse led educational intervention. Holistic follow up including education on inhaler technique and trigger avoidance and self management |
| Who provided the inhaler training? | Nurse |
| Concept | Teach back |
| Measured outcomes | Inhaler technique (via checklist) + PAQLQ and FEV1 |
| Key findings | Inhaler technique improved more in the intervention group but was reverting back to mean by 6 month follow up |

**References**

1. Ammari WG, Al-Hyari N, Obeidat N, Khater M, Sabouba A, Sanders M. Mastery of pMDI technique, asthma control and quality-of-life of children with asthma: A randomized controlled study comparing two inhaler technique training approaches. Pulmonary Pharmacology & Therapeutics. 2017;43:46-54.

2. Agertoft L, Pedersen S. Importance of training for correct Turbuhaler use in preschool children. Acta paediatrica. 1998;87(8):842‐7.

3. Arumugom A, Chandrasekaran V. A randomized comparison between video demonstration and verbal instruction in improving rota haler technique in children with persistent asthma: a pilot study. Journal of clinical and diagnostic research. 2017;11(6):SC05‐SC7.

4. Martin MA, Mosnaim GS, Olson D, Swider S, Karavolos K, Rothschild S. Results from a community-based trial testing a community health worker asthma intervention in Puerto Rican youth in Chicago. Journal of asthma. 2015;52(1):59‐70.

5. Carpenter DM, Alexander DS, Elio A, DeWalt D, Lee C, Sleath BL. Using Tailored Videos to Teach Inhaler Technique to Children With Asthma: Results From a School Nurse-Led Pilot Study. Journal of Pediatric Nursing-Nursing Care of Children & Families. 2016;31(4):380-9.

6. Chan DS, Callahan CW, Sheets SJ, Moreno CN, Malone FJ. An Internet-based store-and-forward video home telehealth system for improving asthma outcomes in children. American journal of health-system pharmacy. 2003;60(19):1976‐81.

7. Grover C, Goel N, Armour C, Van Asperen PP, Gaur SN, Moles RJ, et al. Medication education program for Indian children with asthma: a feasibility stud. Nigerian journal of clinical practice. 2016;19(1):76‐84.

8. Horner SD, Fouladi RT. Improvement of rural children's asthma self-management by lay health educators. Journal of school health. 2008;78(9):506‐13.

9. Kamps AWA, Brand PLP, Roorda RJ. Determinants of correct inhalation technique in children attending a hospital-based asthma clinic. Acta Paediatrica. 2002;91(2):159-63.

10. Kamps AWA, van Ewijk B, Roorda RJ, Brand PLP. Poor inhalation technique, even after inhalation instructions, in children with asthma. Pediatric Pulmonology. 2000;29(1):39-42.

11. Khan R, Yasin F, O'Neill S, Cahalane E, O'Shea R, Browne B, et al. DVD versus physiotherapist-led inhaler education: a randomised controlled trial. Irish medical journal. 2018;111(2):694‐.

12. Minai BA, Martin JE, Cohn RC. Results of a physician and respiratory therapist collaborative effort to improve long-term metered-dose inhaler technique in a pediatric asthma clinic. Respiratory Care.49(6):600-5.

13. Patterson EE, Brennan MP, Linskey KM, Webb DC, Shields MD, Patterson CC. A cluster randomised intervention trial of asthma clubs to improve quality of life in primary school children: the School Care and Asthma Management Project (SCAMP). Archives of disease in childhood. 2005;90(8):786‐91.

14. Root J, Small L. Improving Asthma Control in Children Using the Teach-To-Goal Method. Pediatric Nursing. 2019;45(5):250-7.

15. Shaw N, Souëf P, Turkovic L, McCahon L, Kicic A, Sly P, et al. Pressurised metered dose inhaler-spacer technique in young children improves with video instruction. European Journal of Pediatrics. 2016;175(7):1007-12.

16. Shields M, Alqahtani F, Rivey M, McElnay J. Using remote directly observed therapy (R-DOT) for optimising asthma therapy. European respiratory journal. 2017;50.

17. Turkeli A, Yilmaz O, Yuksel H. Metered dose inhaler-spacer use education effects on achieve asthma control in children. Tuberkuloz ve Toraks.64(2):105-11.

18. Christiansen SC, Martin SB, Schleicher NC, Koziol JA, Mathews KP, Zuraw BL. Evaluation of a school-based asthma education program for inner-city children. Journal of Allergy and Clinical Immunology. 1997;100(5):613-7.

19. Salisbury C, Francis C, Rogers C, Parry K, Thomas H, Chadwick S, et al. A randomised controlled trial of clinics in secondary schools for adolescents with asthma. Br J Gen Pract. 2002;52(485):988-96.

20. Schultz A, Sly PD, Zhang G, Venter A, Le Souëf PN, Devadason SG. Incentive device improves spacer technique but not clinical outcome in preschool children with asthma. Journal of paediatrics and child health. 2012;48(1):52-6.

21. Aziz NA, Norzila MZ, Hamid MZ, Noorlaili MT. Skills amongst parents of children with asthma: a pilot interventional study in primary care setting. Medical Journal of Malaysia.61(5):534-9.

22. Bynum A, Hopkins D, Thomas A, Copeland N, Irwin C. The effect of telepharmacy counseling on metered-dose inhaler technique among adolescents with asthma in rural Arkansas. Telemedicine journal and e-health. 2001;7(3):207‐17.

23. Foland AP, Stern T, Ramacciotti T, Martin J, Gilbert I, Cohn RC. Improvement of metered-dose inhaler administration technique: The effect of training sessions at a specialized pediatric asthma compliance and technique clinic. Current Therapeutic Research-Clinical and Experimental. 2002;63(2):142-7.

24. Horner SD, Brown A. Evaluating the effect of an asthma self-management intervention for rural families. Journal of asthma. 2014;51(2):168‐77.

25. Sleath B, Carpenter DM, Ayala GX, Williams D, Davis S, Tudor G, et al. Communication during pediatric asthma visits and child asthma medication device technique 1 month later. Journal of Asthma.49(9):918-25.

26. Volerman A, Toups MM, Hull A, Press VG. A feasibility study of a patient-centered educational strategy for rampant inhaler misuse among minority children with asthma. Journal of Allergy and Clinical Immunology-in Practice. 2019;7(6):2028-30.

27. Gracia-Antequera M, Morales Suarez-Varela M. An intervention to improve the inhalatory technique of children and adolescents with asthma. Allergologia et Immunopathologia.27(5):255-60.

28. Sirimontakan T, Manuyakorn W, Wattanarungsun P, Thongkum K, Chonpaisan K, Kamalaporn H. Efficacy of a Newly-Developed Cartoon Video Instruction to Improve Dry Powder Inhaler Technique in Thai Children with Asthma. American Journal of Respiratory and Critical Care Medicine. 2019;199.

29. Jové Blanco, Ana, et al. "Impact of an Asthma Education Program During Admission." *Hospital Pediatrics* 11.8 (2021): 849-855.

30. Azak, Merve, Birsen Mutlu, and Zeynep Tamay. "The effect of training of metered-dose inhaler technique on asthma control and quality of life in children with asthma: a randomized trial." *Children's Health Care* 50.1 (2021): 44-63.

31. Kapoor, I., R. Aulakh, and S. Randev. "VIDEO EDUCATION VS PHYSICAL DEMONSTRATION OF INHALER TECHNIQUE FOR CHILDREN WITH ASTHMA: A RANDOMIZED CONTROLLED TRIAL." *Chest* 157.6 (2020): A200.

32. Tony, Sara M., et al. "The effect of adding a training device and smartphone application to traditional verbal counseling in asthmatic children." *International Journal of Clinical Practice*(2021): e14401.

33. Gao, Guozhen, et al. "A randomized controlled trial of a nurse‐led education pathway for asthmatic children from outpatient to home." *International journal of nursing practice* 26.3 (2020): e12823.
